# On the *vis inertiæ* within burnout research

**DOI:** 10.1308/rcsann.2025.0032

**Published:** 2025-04-11

**Authors:** R Brisson

**Affiliations:** University of Luxembourg

Chahal and Matwala recently published an article entitled ‘A systematic review of the prevalence of burnout in orthopaedic surgeons’ in the *Annals*.^1^ The authors reviewed 34 studies and found burnout prevalence to range from 15% to 90%. The Maslach Burnout Inventory (MBI) was used in 73.5% of the examined body of research. Burnout was assessed in the remaining studies with the abbreviated MBI, the Mini Z 2.0, the Oldenburg Burnout Inventory, the Physician Well-Being Index, the Professional Fulfillment Index, the Well-Being Index and a burnout questionnaire created by the American Public Welfare Association. The study conclusions stressed the large burnout prevalence in orthopaedic surgeons (i.e. about 50%) and advocated for the implementation of interventions that ‘prevent burnout from irrevocably damaging the future of orthopaedic surgery’.^1^^,p.61^ In my view, these conclusions are problematic for at least four reasons.

First, the authors seem to ignore that burnout researchers have failed to provide sound diagnostic criteria of the condition.^2–4^ Consequently, the methods employed to identify burned-out individuals in the reviewed studies are arbitrary. Because investigators are unable to distinguish between burned-out and non-burned-out individuals, estimating burnout prevalence is aporetic. This state of affairs questions the very foundation of Chahal and Matwala’s review.

Second, the authors overlooked the heterogeneity of the definitions of burnout involved in the reviewed studies. For example, the tacit view that the Oldenburg Burnout Inventory, which measures exhaustion and disengagement, and the Mini Z 2.0, which asks participants to rely on their own definition of burnout, assess the same phenomenon is, to say the least, disputable. All in all, it is unclear what burnout exactly reflects in the authors’ study.

Third, the authors took Maslach’s tripartite definition of burnout as a point of reference, a definition also adopted by the World Health Organization.^4,5^ The authors thus depicted the phenomenon as a *syndrome*, that is, a set of *concurrent* symptoms involving, here, exhaustion, depersonalisation (or cynicism) and lack of accomplishment (or inefficacy). However, most of the reviewed studies used the MBI and considered as burned-out any individual exhibiting a given score on either the exhaustion subscale *or* the depersonalisation subscale.^6^ This practice misaligns with the view that burnout is a syndrome and reflects a contradiction between the conceptualisation and the assessment of burnout. As noted elsewhere, such a mismatch originates in the MBI manual.^7,8^ Although the manual presents burnout as a syndrome, it invites investigators to approach each core element of burnout separately. As a result, most of the studies reviewed by Chahal and Matwala assessed distinct entities (i.e. exhaustion, depersonalisation and lack of accomplishment) rather than their syndromic combination (i.e. burnout).

Fourth, most reviewed studies employed cut-offs available in the MBI manual to distinguish between cases and non-cases. Although widely used since their publication in the third edition of the MBI manual in 1996, these cut-offs have been described recently as ‘arbitrary’ and a ‘mistake’ by the MBI developers.^9,10^ Given the abovementioned limitations and the poor psychometric properties of the instrument, this belated volte-face raises questions about the continued MBI commercialisation and use.^11^ It also questions the validity of the prevalence estimates documented in the studies relying on the MBI and, by implication, Chahal and Matwala’s review itself.

All things considered, I recommend suspending investigations into burnout prevalence until burnout is clearly and validly diagnosable. Despite decades of extensive research, the flaws affecting the burnout construct remain unresolved.^12,13^ The reasons for this inertia require clarification, as it has involved numerous unsound publications and led to misallocation of human and financial resources.
